# Efficiency of novel nanocombinations of bovine milk proteins (lactoperoxidase and lactoferrin) for combating different human cancer cell lines

**DOI:** 10.1038/s41598-017-16962-6

**Published:** 2017-12-01

**Authors:** Marwa M. Abu-Serie, Esmail M. El-Fakharany

**Affiliations:** 1 Medical Biotechnology Department, Genetic Engineering and Biotechnology Research Institute, City for Scientific Research and Technology Applications (SRTA-City), New Borg EL-Arab, 21934 Alexandria, Egypt; 2 Protein Research Department, Genetic Engineering and Biotechnology Research Institute, City for Scientific Research and Technology Applications (SRTA-City), New Borg EL-Arab, 21934 Alexandria, Egypt

## Abstract

Bovine lactoperoxidase (LPO) and lactoferrin (LF) are suitable proteins to be loaded or adsorbed to chitosan nanoparticles (NPs) for preparing stable nanoformulations with potent anticancer activity. In the present study, nanocombinations of LPO and LF revealed improvement in their stability and activity compared to single (free or nanoformulated) bovine proteins. The coating or loading of LPO-loaded NPs with LF resulted in the highest synergistic cytotoxicity effect against Caco-2, HepG-2, MCF-7 and PC-3 cells in comparison with other NPs and free proteins without causing toxicity toward normal cells. This synergistic improvement in the anticancer activity was apoptosis-dependent that was confirmed by severe alterations in cellular morphology, high percentage of annexin-stained cells and sub-G1 populations as well as nuclear staining with orange fluorescence of treated cancer cells. Additionally, significant alterations in the expression of well characterized cellular proliferation and apoptosis guards (NF-κB, Bcl-2 and p53) in these NPs-treated cancer cells compared to 5-fluorouracil (5-FU) treated cells. Our findings provide for the first time that these new synergistic nanoformulated forms of LPO and LF were superior in their selective apoptosis-mediating anticancer effect than free form of these proteins and 5-FU. LF coating or loading of LPO-loaded NPs present as promising therapy for cancer.

## Introduction

Bovine milk is a precursor of different biologically active anticancer proteins. Although whey-contained proteins represent the minor part of bovine milk, it exhibited range of biological activities^1,2^. The most important active proteins of whey are α-lactalbumin (α-LA), lactoperoxidase (LPO) and lactoferrin (LF) are known to play multi-functional and biological roles^3–5^.

Lactoperoxidase is one of the most crucial whey enzymes that are able to form potent biocidal small molecules by oxidizing halides and pseudohalides using hydrogen peroxide. This hydrogen peroxide is actually destructive to the epithelium and its level needs to be tightly controlled. Previous studies have reported that the LPO system has a role for in the preservation of raw milk, in airway defense and broad biocidal activity against pathogenic microorganisms^6–8^. However, LPO shows antioxidant activity and exerts ability to degrade carcinogenic compounds^9,10^. Its tumoricidal activity has only seldom been reported elsewhere. LF is an iron binding protein with many relevant biological functions including antimicrobial activity, antioxidant properties, anti-inflammatory activity and protection function against cancer development and metastasis^11,12^. The iron-saturated form of LF (hololactoferrin) and its derived peptides have also been demonstrated to be competent anticancer drugs^13,14^. There are several *in vitro* and *in vivo* studies revealed that LF and its derived peptides can inhibit the growth of tumors^13–16^.

Herein, we investigated the increment in anticancer activity of LPO before and after mixing with LF and nanoformulating using chitosan. Chitosan nanoparticles (NPs) exhibit multiple physical, chemical and biological properties such as readiness to be modified, biodegrability, biocompatibility, non-toxicity and muco-adhesiveness. Therefore, they are used to improve the stability and efficacy of many drugs including genes, anticancer compounds and antibiotics^17^. Thus, chitosan NPs have been used as promising carriers for therapeutic proteins which still have obstacles in delivery at their standard pharmacodynamics due to instability and their nature which hampers transport through cellular membrane^18,19^. In addition, proteins adsorption and interaction with NPs has become the subject of intense investigation and the basis of NPs bio-reactivity^19^. In general, proteins binding to NPs can lead to the loss of secondary structure and consequent changes in the proteins activity which can be considered as a limitation of NP efficacy but there is a potential positive side to induce intense properties on the protein interactions and stability^18,20^.

Therefore it is necessary to evaluate anticancer efficacy of these milk proteins before and after nanocombinations against the most common and virulent cancers (colon, liver, breast and prostate). This anticancer potential was evaluated by detecting the dose of growth inhibition, percentage of apoptosis and alterations in morphology, cell cycle as well as in expression of apoptosis-related genes in the studied cancer cell lines.

## Results

### Characterization of the purified LPO and/or LF-loaded/coated to chitosan NPs

**Figure 1:**
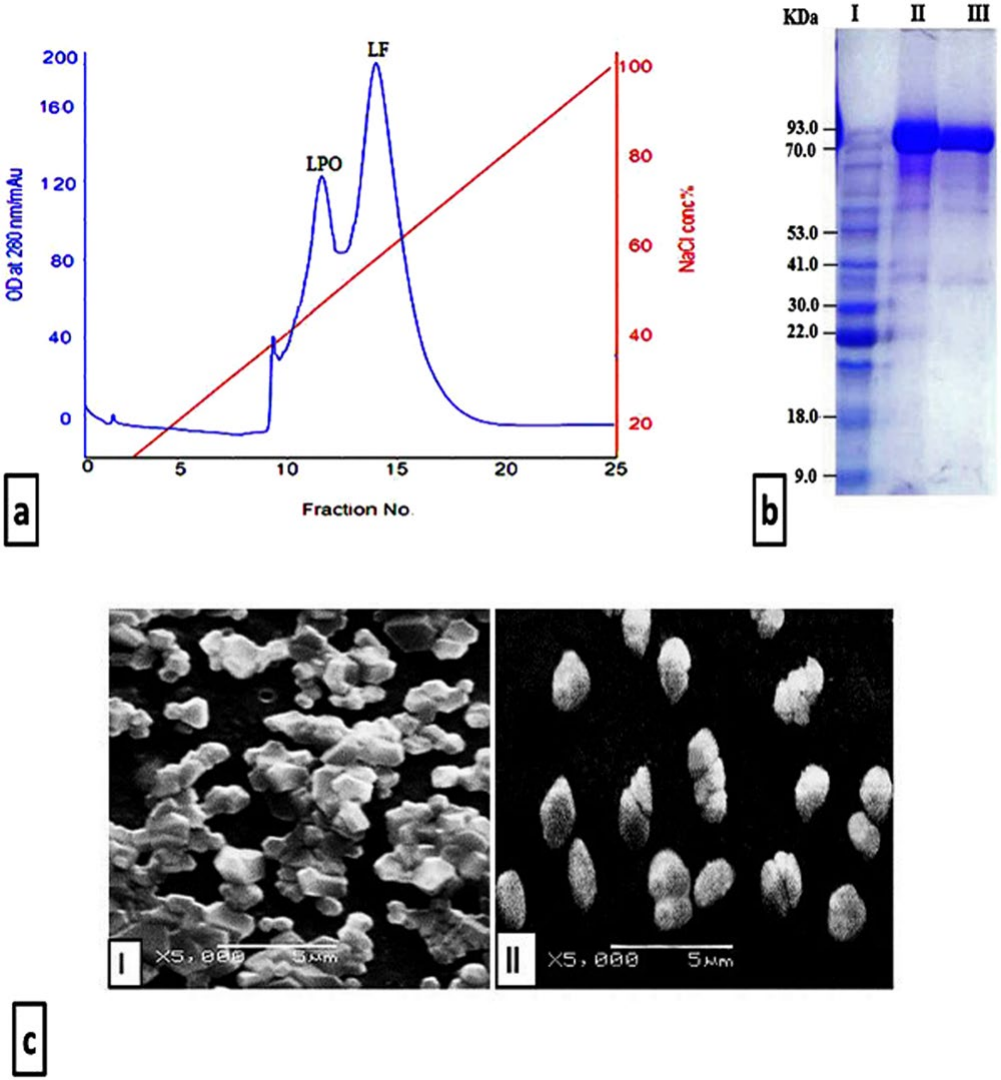
Purification of LPO and LF and scanning electron micrograph of the most active LPO and LF NPs. (**a**) Elution profile of LPO and LF on a Mono S column. (**b**) 12% SDS-PAGE for bovine LPO and LF; Lane I is protein marker, lane II is purified LF and lane III is is purified LPO. (**c**) Morphology of the most active NPs (I) LPO + LF-loaded NPs and (II) LF coated LPO-loaded NPs.

Skimmed bovine milk was applied to a Mono S column and both LPO and LF were eluted at NaCl gradient of 0.4–6.0 M ad 0.6–0.8 M, respectively (Fig. 1a). The peaks containing LPO or LF were concentrated and applied separately to Sephacryl S200 column. Homogeneity of the two purified proteins was visualized by 12% SDS-PAGE and both corresponded to a molecular weight of ~78 kDa and~78 kDa for LPO ad LF, respectively (Fig. 1b).

After preparation of loaded LPO and LF NPs, their percentages of LC and Encaps were more than 58% and 88%, respectively as shown in Table 1. The Des percentage of LPO was about 76% during coating LPO on free NPs or LF-loaded NPs and about 97% during coating LF on free NPs or LPO-loaded NPs, while Des value for coating both LPO and LF on free NPs was 94.8% (Table 1). Table 1 demonstrates that the size of LPO-loaded NPs, LF-loaded NPs and LPO + LF-loaded NPs ranged from 214 to 389 nm with zeta potential measurement around 51 mV whereas the size of LPO coated NPs and LF coated NPs were 297 and 323.5 nm, respectively, with zeta potential of 16.6 and 17.7 mV, respectively. However, the size of LPO + LF coated NPs, LF coat LPO-loaded NPs and LPO coated LF-loaded NPs were increased to be more than 460.5 nm and the zeta potential measurements were decreased to be 10.9, 20.6 and 19.9 mV, respectively (Table 1). The morphology of the prepared LPO + LF-loaded NPs and LF coated LPO-loaded NPs was illustrated in Fig. 1c.

**Table 1:** Encapsulation efficiency, loading and desorption capacity, size and zeta potential of the prepared NPs.

Sample	%LC	% Encaps	%Des	Size (nm)	Zeta potential (mV)
LPO-loaded NPs	62.49 ± 0.09	88.9 ± 0.12	—	214.7 ± 16	50.5
LPO coated NPs	—	—	76.68 ± 0.8	297 ± 8	16.8
LF-loaded NPs	64.38 ± 0.62	91.6 ± 0.88	—	228.5 ± 9.5	51
LF coated NPs	—	—	96.84 ± 0.65	323.5 ± 7.5	17.7
LPO + LF-loaded NPs	58.5 ± 0.56	83.29 ± 0.8	—	389 ± 11	51.4
LPO + LF coated NPs	—	—	94.78 ± 0.22	507 ± 18	10.9
LF coat LPO-loaded NPs	62.3 ± 0.92	88.7 ± 1.3	96.7 ± 0.3	477.5 ± 12.5	20.6
LPO coated LF-loaded NPs	64.18 ± 0.37	91.3 ± 0.53	75.6 ± 0.65	460.5 ± 10.5	19.9

All values were expressed as mean ± SEM.

### LPO and LF stabilities before and after nanoformulations

**Figure 2:**
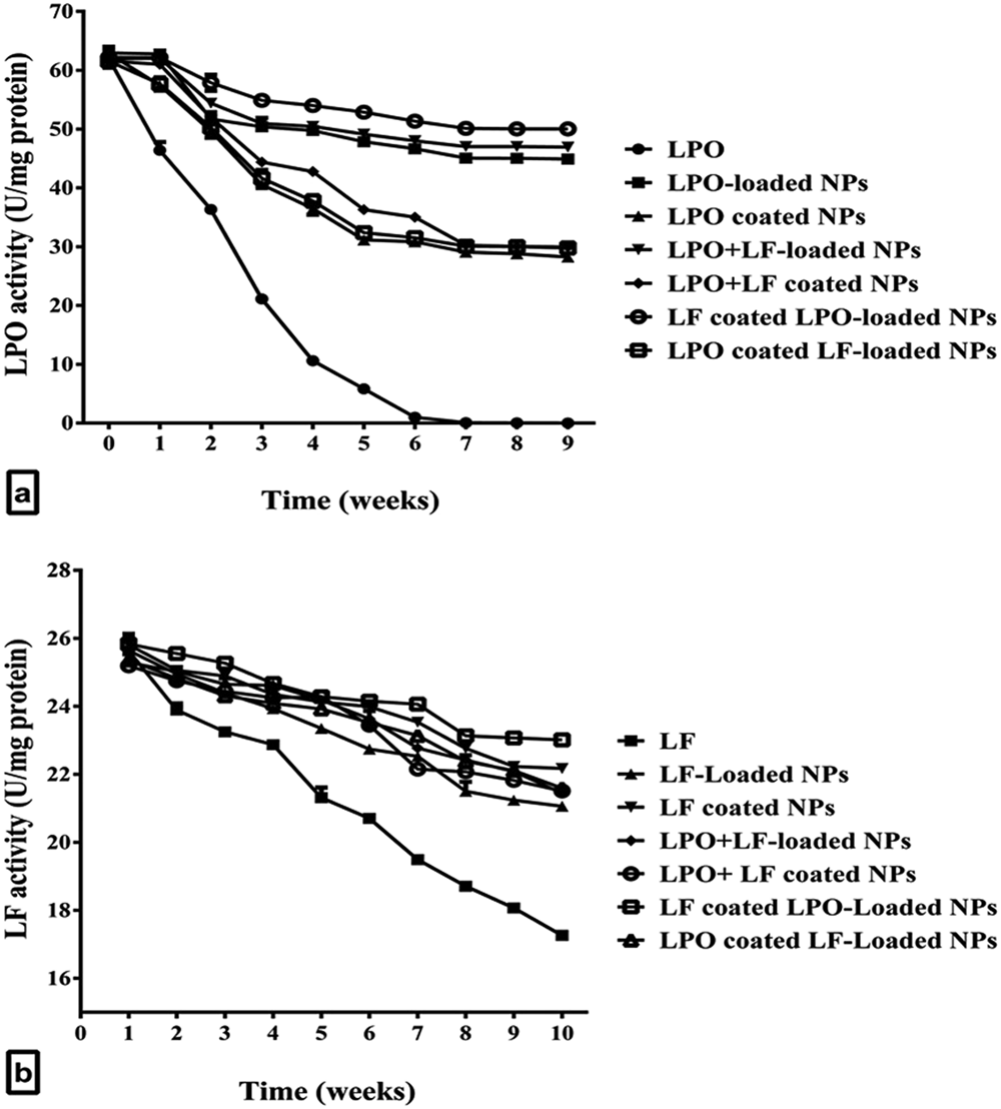
Stability of bovine milk protein activities as free and nanoformulated forms throughout 9 weeks. (**a**) LPO. (**b**) LF.

The stabilities of LPO and LF in all eight nanoformulated forms and their free form were detected through estimation activities of LPO and LF during storage condition at 4 °C for 9 weeks. In terms of stability, the stored free LPO lost its activity gradually by increasing the storage time; the activity became 36% of its initial activity after 3 weeks and was lost completely after 7 weeks of storage at 4 °C. Activities of the LPO coated to chitosan NPs (LPO coated NPs, LPO + LF coated NPs and LPO coated LF-loaded NPs) decreased to become around 45%–48% after 9 weeks storage. However, LPO that was loaded to chitosan NPs lost only about 28% of their initial activities under the same conditions of storage (Fig. 2a). It is important to note that the loaded LPO which was combined with LF, by loading or coating, retained about 75.8% to 80.4% of its initial activity after 9 weeks of storage at 4 °C (Fig. 2a). Accordingly, the loaded forms of LPO were more able to retain high LPO activity, particularly when loaded or coated with LF, than its coated forms that possessed only 48.5% of LPO activity. In contrast to LPO, free LF retained its activity after storage in the same conditions for long time and lost 32% of its initial activity after 9 weeks (Fig. 2b). Also, Fig. 2b demonstrates that all nanoformulated LF retained more than 82% of its initial activity in control with 67.3% for free LF. These indicated that the nanoformulation of LPO and LF possessed stable activities compared to those of free protein samples for 9 weeks at 4 °C, particularly, LPO-, LPO + LF-loaded NPs and LF coated LPO-loaded NPs as well as LF-coated NPs.

### Cytotoxicity of free and nanoformulating LPO and LF toward human normal cells

**Figure 3:**
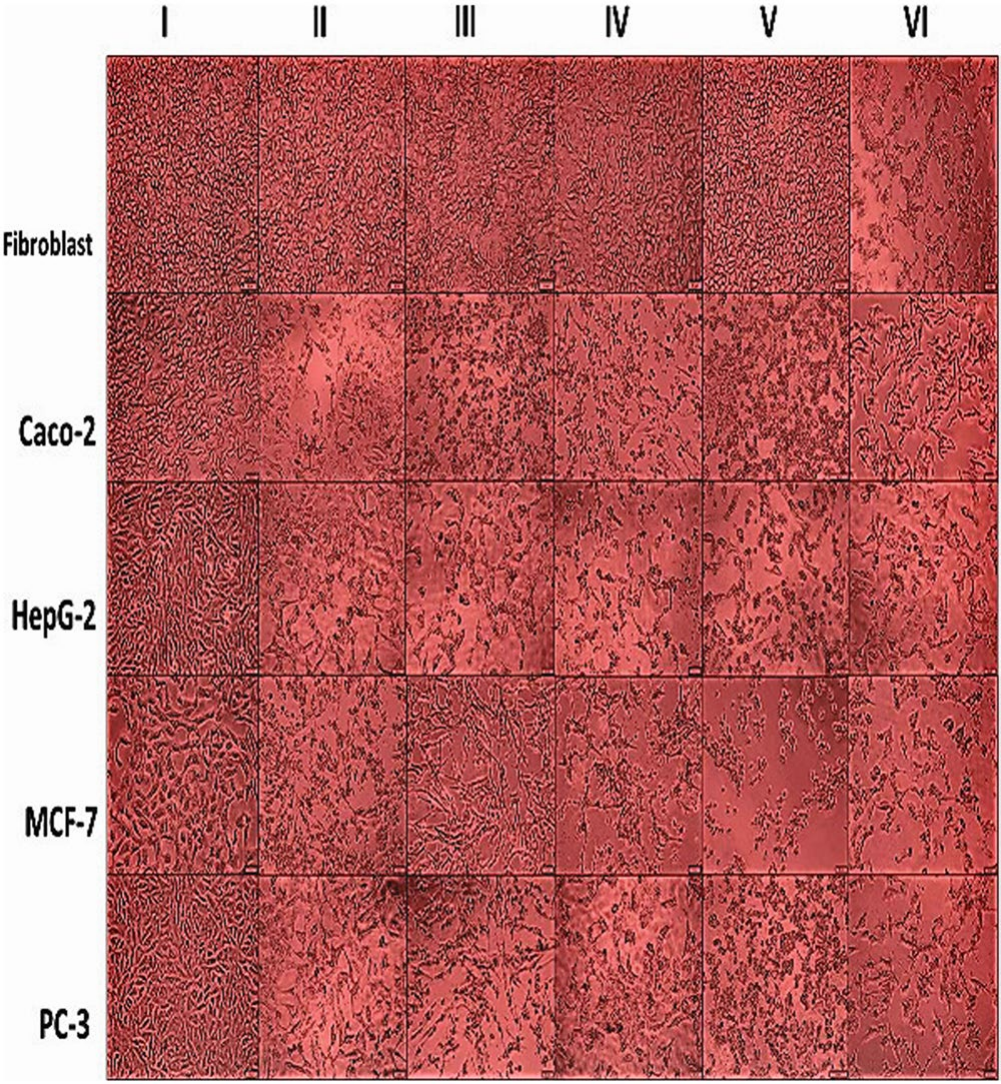
Normal and cancer cell morphology shown by phase contrast microscope. Human cancer cell lines (I) including fibroblast cells, Caco-2 cells, HepG-2 cells, MCF-7 cells and PC-3 cells following 72 h treatment with (II) LPO-loaded NPs, (III) LF-coated NPs, (IV) LPO + LF-loaded NPs, (V) LF coated LPO-loaded NPs and (VI) 5-FU.

The viability of fibroblast cells was evaluated after treatment with free purified proteins and loaded or coated proteins to chitosan NPs in control with 5-FU, the highest IC_50_ and EC_100_ values refer to the highest safety. As given in Supplementary Table S1, the safe dose (EC_100_) for all tested samples (315–1388 µg/ml) were significantly higher than 5-FU (0.8 µg/ml). Both IC_50_ and EC_100_ values for LPO-and/or LF-loaded or coated to chitosan NPs (≥1000 and 334 µg/ml, respectively) were found to be higher than free or combined LPO and LF (<902 and 316 µg/ml, respectively). The safety of the most effective NPs towards a normal human fibroblast cell line was demonstrated in Fig. 3.

### Antitumor effect of free and nanoformulating LPO and LF on different human cancer cell lines

Table 2 reveals that all loaded or coated NPs had an anticancer effect on all tested cancer cell lines at IC_50_ lower than that of their corresponding free protein samples. Based on the lowest IC_50_ value refers to the highest anticancer activity, LPO-loaded NPs showed the highest toxic effect against all cell lines than LPO coated NPs in contrast to LF-loaded NPs which exhibited the lowest toxic effect against all cell lines than LF coated NPs. Whereas LPO + LF-loaded NPs and coated LPO-loaded NPs exerted significantly higher toxic effect against cancer cells than LPO + LF coated NPs and LPO coated LF-loaded NPs (Table 2). MCF-7 cells were the most sensitive cells for treatment with NPs, mostly LF-coated LPO-loaded NPs. Table 2 illustrates that HepG-2 cell line is the next sensitive cell line for NPs treatment and LF-coated LPO-loaded NPs showed the highest activity against these cells. Meanwhile LPO + LF-loaded NPs significantly exhibited high toxicity against PC-3 cells and Caco-2 cells. The four NPs (LPO-loaded NPs, LF-coated NPs, LPO + LF-loaded NPs and LF coated LPO-loaded NPs) exerted a significant potent anticancer effect with lower IC_50_ than that (IC_50_ > 546) of free LPO, free LF and LPO + LF against all tested cancer cell lines without causing toxicity to normal cells (SI > 3.1). On the other hand, 5-FU (currently used chemotherapy) showed a high toxic effect against all tested cells, both cancer and normal cells with the lowest SI values (<1.74) that illustrated lack of 5-FU selectivity between normal cells and cancer cells.

**Table 2:** IC_50_ (µg/ml) and SI values of all tested NPs samples against human cancer cell lines.

Sample	Caco-2		HepG-2		MCF-7		PC-3	
	IC_50_	SI	IC_50_	SI	IC_50_	SI	IC_50_	SI
Free NPs	1989.8 ± 13	0.22 ± 0.004	432.5 ± 9.5	3.18 ± 0.89	925.2 ± 10.8	0.47 ± 0.007	736.9 ± 19.6	1.87 ± 0.061
LPO	2029.4 ± 25	0.41 ± 0.009	873.9 ± 7.4	0.95 ± 0.03	1926.9 ± 17	0.43 ± 0.018	1154.3 ± 12	0.72 ± 0.02
LPO-loaded NPs	813.1 ± 60.3	3.15 ± 0.01	503.2 ± 20.2	5.1 ± 0.02	339.7 ± 4.5	7.5 ± 1.6	776.8 ± 7.9	3.29 ± 0.015
LPO coated NPs	1496.3 ± 11	0.55 ± 0.01	703.8 ± 52	1.72 ± 0.12	374.7 ± 7.8	2.4 ± 0.03	847.2 ± 14.2	1.43 ± 0.1
LF	2282.2 ± 21	0.36 ± 0.006	636.2 ± 21	1.28 ± 0.02	1955.6 ± 37	0.42 ± 0.003	874.7 ± 6.3	0.93 ± 0.01
LF-loaded NPs	1285.2 ± 59	1.06 ± 0.04	510.6 ± 15	2.66 ± 0.98	269.7 ± 6.2	5.03 ± 0.19	812.2 ± 4.5	1.67 ± 0.06
LF coated NPs	755.1 ± 84	3.06 ± 0.11	463 ± 29	5.0 ± 0.18	189.1 ± 7.2	12.26 ± 0.44	605.2 ± 21.4	3.82 ± 0.14
LPO + LF	1980.9 ± 13	0.45 ± 0.008	545.6 ± 18	1.65 ± 0.02	1846.6 ± 7.7	0.49 ± 0.008	730.1 ± 4.4	1.23 ± 0.016
LPO + LF-loaded NPs	816.6 ± 42	4.02 ± 0.42	472.8 ± 15	6.94 ± 0.07	215.9 ± 3	15.21 ± 0.16	620.3 ± 17	5.3 ± 0.057
LPO + LF coated NPs	1173.9 ± 70	0.94 ± 0.05	540.5 ± 8	2.04 ± 0.1	713.6 ± 15.5	1.54 ± 0.081	811.8 ± 38	1.38 ± 0.07
LF coated LPO-loaded NPs	669.2 ± 40	3.16 ± 0.02	256.9 ± 38	8.23 ± 0.05	150.1 ± 4.8	14.1 ± 0.093	539.3 ± 29	3.92 ± 0.026
LPO coated LF-loaded NPs	968.4 ± 77	1.04 ± 0.11	542.7 ± 29	1.8 ± 0.02	244.7 ± 14	4.1 ± 0.26	815.5 ± 42	1.23 ± 0.07
5-FU	1.09 ± 0.02	1.74 ± 0.1	1.23 ± 0.05	1.53 ± 0.05	1.60 ± 0.05	1.19 ± 0.12	1.46 ± 0.04	0.72 ± 0.02

All values were expressed as mean ± SEM.

The above data were supported by an obvious damage in the morphology of all treated human cancer cells at IC_50_ concentrations of the most effective NPs (LPO-loaded NPs, LF-coated NPs, LPO + LF-loaded NPs and LF coated LPO-loaded NPs) in comparison with healthy untreated cancer cells as negative control as shown in Fig. 3. Additionally, the high selectivity (SI) of these most effective NPs was confirmed by non-obvious morphological changes between untreated and NPs-treated normal human fibroblast cells. However, severe alteration in the morphology of 5-FU treated-normal and cancer cells was observed as sign of its low selectivity (Fig. 3).

There is a synergism (CI < 1) between LPO and LF that was observed by the values of CI (0.9 ± 0.003, 0.83 ± 0.08, 0.95 ± 0.02 and 0.75 ± 0.02) of LPO + LF for its anticancer activity toward Caco-2, HepG-2, MCF-7 and PC-3 cell lines, respectively. Additionally, a synergistic anticancer effect was recorded between chitosan, LPO and LF in single and combined NPs based on the estimated CI values (0.71 to 0.94) excluding LPO coated NPs, LPO + LF-coated NPs and LPO coated LF-loaded NPs had antagonistic effects (CI > 1). The strongest synergy (CI ≤ 0.8) in LF coated LPO-loaded NPs and LPO + LF-loaded NPs was followed by CI values (CI ≤ 0.9) of LF-coated NPs, LF-loaded NPs and LPO-loaded NPs for their cytotoxicity against cancer cell lines (Supplementary Table S2). Accordingly, these CI data declared that LPO adsorption on unloaded NPs or LF-loaded chitosan NPs did not result in positive effect on LPO activity (CI > 1) while LPO loading was able to sustain LPO activity thus the net effect was synergistic (CI < 1). On the other hand, loading or coating of LF on free or LPO-loaded chitosan NPs revealed a positive effect (CI < 1).

### Apoptosis-dependent antitumor activity of LPO and/or LF-loaded/coated to chitosan NPs

**Figure 4:**
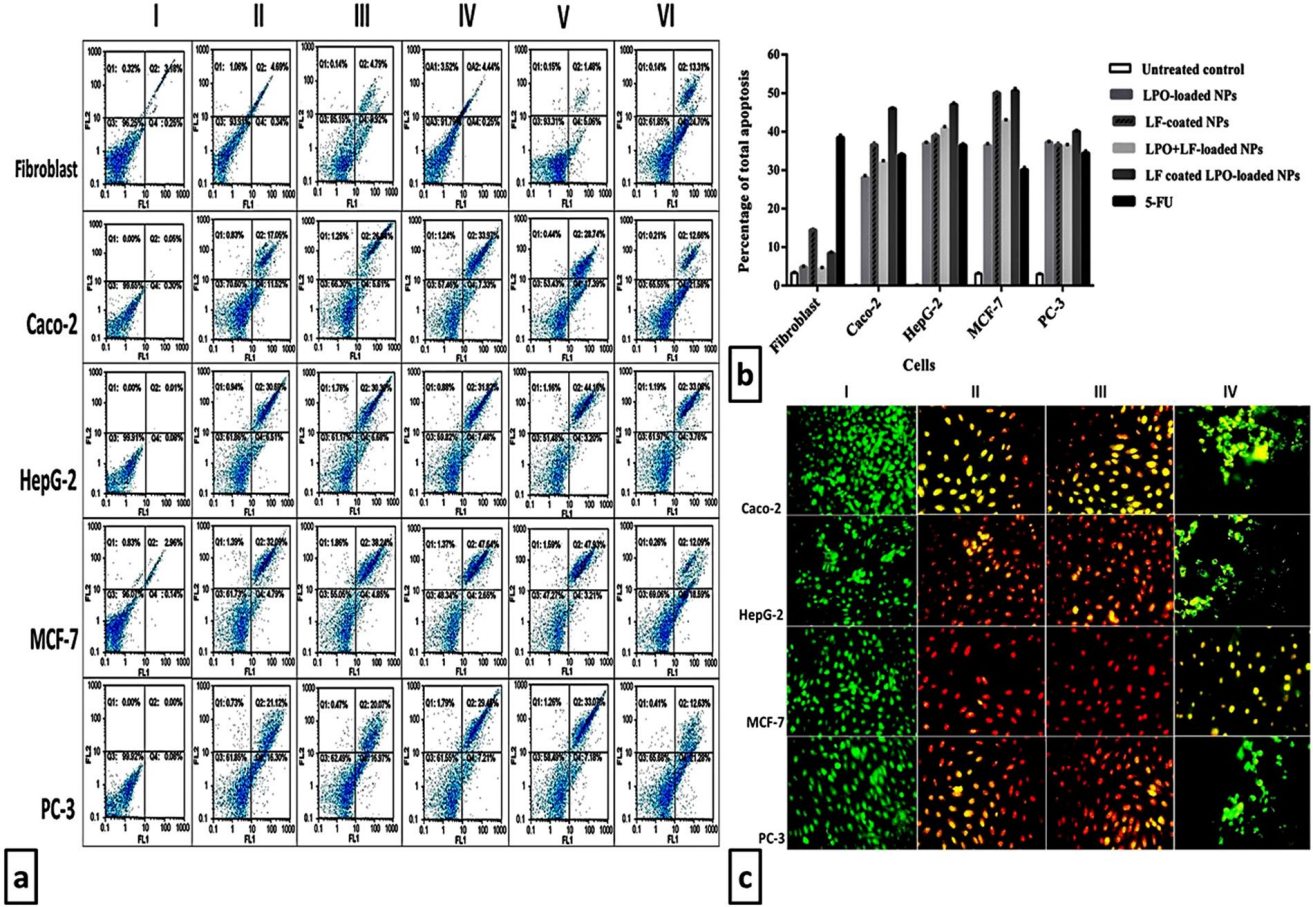
Investigation of the apoptotic effect of the most effective NPs using double nuclear staining. (**a**) Flow charts of annexin-PI analysis of cancer cells lines (I) including fibroblast cells, Caco-2 cells, HepG-2 cells, MCF-7 cells and PC-3 cells following 72 h treatment with (II) LPO-loaded NPs, (III) LF-coated NPs, (IV) LPO + LF-loaded NPs, (V) LF coated LPO-loaded NPs and (VI) 5-FU. (**b**) The percentage of total apoptosis in NPs-treated normal and cancer cells in comparison with untreated cells. (**c**) Fluorescence images of ethidium bromide-acridine orange staining of cancer cells lines (I) including Caco-2 cells, HepG-2 cells, MCF-7 cells and PC-3 cells following 72 h treatment with (II) LPO + LF-loaded NPs, (III) LF coated LPO-loaded NPs and (IV) 5-FU.

The apoptotic effect of LPO and/or LF-loaded/coated to chitosan NPs at IC_50_ doses on human cancer cells was assessed using a double staining annexin-V/PI in control with IC_50_ of 5-FU (Fig. 4a,b). Our results showed that MCF-7 cells followed by HepG-2 cells were the most sensitive to IC_50_ doses of coated or loaded NPs; LF coated LPO-loaded NPs had significant anticancer effect, resulting in more than 47% of cancer cells were apoptotic. LF coated LPO-loaded NPs treated Caco-2 cells and PC-3 cells were the least sensitive cells with the total percentages of apoptosis above 40%. The second active sample was LPO + LF-loaded NPs inducing apoptosis at its IC_50_ in four cancer types by less than 42.9 ± 0.2%. LF-coated NPs, at its IC_50_ doses, are the third most active with the total percentage of apoptosis between 36.6 ± 0.5% and 39 ± 0.3% for all tested cancer cells except MCF-7 which was 50 ± 0.2%. LPO-loaded NPs had less apoptotic effects at IC_50_ values with apoptotic cell population ranging from 28.1 ± 0.4% to 36.5 ± 0.4% and 5-FU had a moderate efficacy to induce the apoptosis either in normal or different cancer cells at IC_50_ doses with apoptosis values higher than 30% (Fig. 4a,b). In contrast, all prepared loaded or coated NPs possessed smaller apoptosis percentage toward normal human cell line below 8.5% except LF-coated NPs which were the highest (14.5 ± 0.2%). This confirmed LF coated LPO-loaded NPs and LPO + LF-loaded NPs were the most significant anticancer NPs, among all (single or combined) prepared NPs and 5-FU, as selective apoptotic inducers in cancer cells (Fig. 4a,b).

Based on CI values (0.77 ± 0.01 & 0.71 ± 0.01, 0.84 ± 0.01 & 0.81 ± 0.001, 0.83 ± 0.01 & 0.86 ± 0.004 and 0.91 ± 0.004 & 0.92 ± 0.003) of the two most effective nanocombinations in term of their total apoptosis percentage of Caco-2, HepG-2, MCF-7 and PC-3, respectively, no significant difference between them was recorded. This assures that both nanocombinations resulted in high synergistic apoptotic effects on cancer cells.

Moreover, AO-EB nuclear staining ascertained the incidence of apoptosis at late stage in LF coated LPO-loaded NPs- and LPO + LF-loaded NPs-treated cancer cell lines that have lost their membrane integrity and emitted orange and red fluorescence instead of green fluorescence in untreated cancer cells (Fig. 4c). While FU-treated cancer cells emitted bright green or yellow that is an indicator of early phase of apoptosis as it was demonstrated in Fig. 4c IV.

### Flow cytometric analysis of cell cycle distribution of LF coated LPO-loaded NPs and LPO + LF-loaded NPs-treated cancer cells

**Figure 5:**
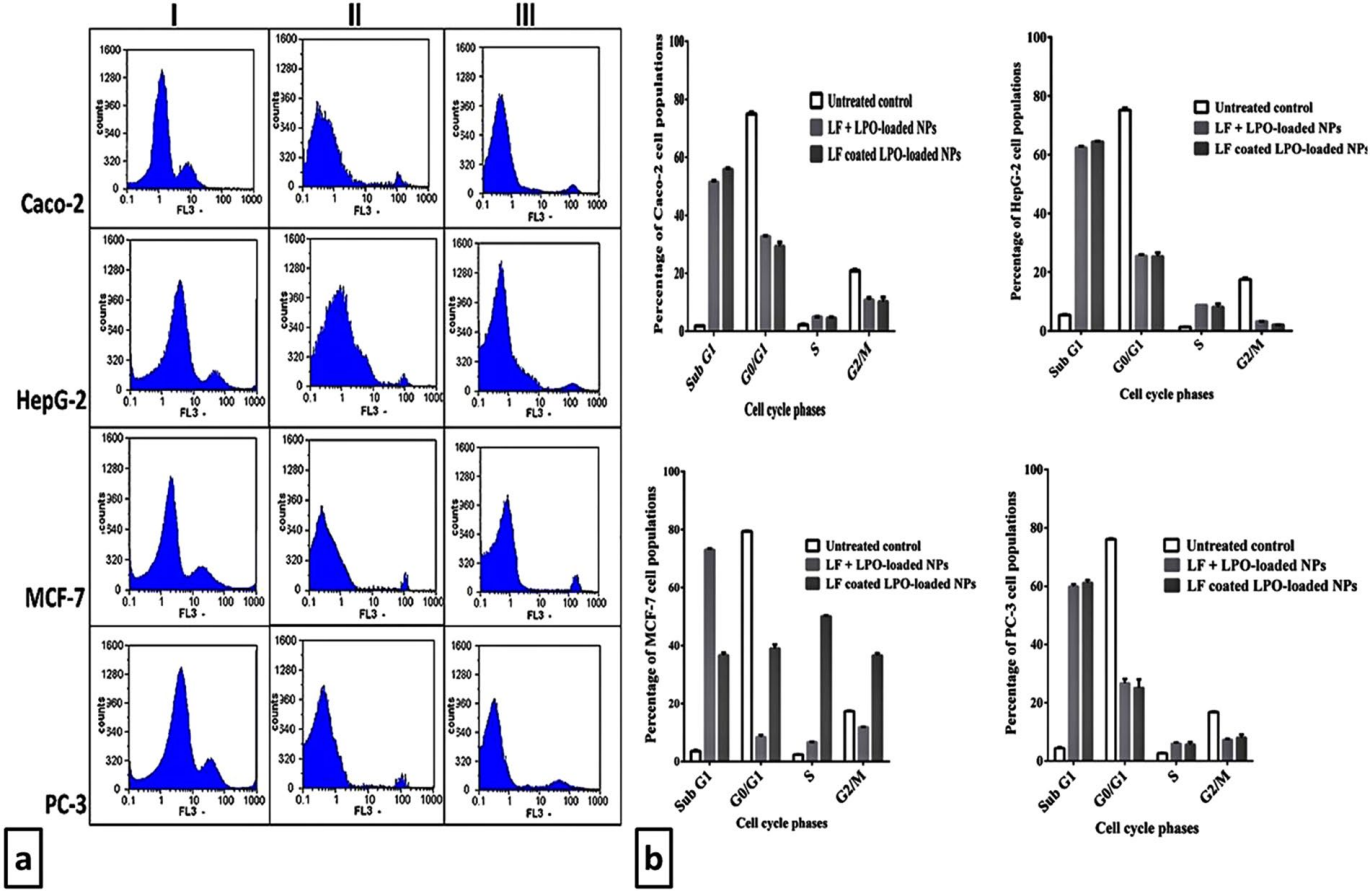
Cell cycle analysis of the two most effective NPs-treated human cancer cell lines. (**a**) Flow charts of cell cycle analysis of human cancer cell lines (I) including Caco-2 cells, HepG-2 cells, MCF-7 cells and PC-3 cells following 72 h treatment with (II) LPO + LF-loaded NPs and (III) LF coated LPO-loaded NPs. (**b**) Quantitative distribution of LPO + LF-loaded NPs- and LF coated LPO-loaded NPs-treated cancer cell populations in different phases of cell cycle in comparison with untreated cancer cells.

Alteration of cell cycle phases was investigated for the two highest effective NPs (LF coated LPO-loaded NPs and LPO + LF-loaded NPs) at their IC_50_ values after 72 h incubation with four human cancerous cells (Fig. 5a,b). Both NPs have the ability to induce cell cycle arrest in the both main check points of cell cycle progression phases (G0/G1 and G2/M phases). After treatment with these NPs, a significant reduction in the peaks of G0/G1 and G2/M phases coupled with increase in sub G1 phases was observed, however S phases were slightly increased as shown in Fig. 5a,b. The percentages of NPs-treated cancer cells in G1 and G2 phases were less than 33% and 12%, respectively compared with that of untreated cancer cells which were around 75% and 17%, respectively phase (Fig. 5b). Figure 5b also shows that NPs-treated MCF-7 cell line was the most sensitive to the increase in the percentage of sub G1 (73% of the cell population), followed by HepG-2 cells (>62% of the cell population), PC-3 cells (>59% of the cell population) and lastly were Caco-2 cells (>51% of the cell population), compared with the controls which ranged from 1.87 ± 0.03 to 5.39 ± 0.3%. In addition, no significant change was observed between LF coated LPO-loaded NPs and LPO + LF-loaded NPs in cell cycle analysis of treated MCF-7 and PC-3 cell lines.

### Effect of the most active anticancer NPs on NF-κB, Bcl-2 and p53 expression in human cancer cell lines

**Figure 6:**
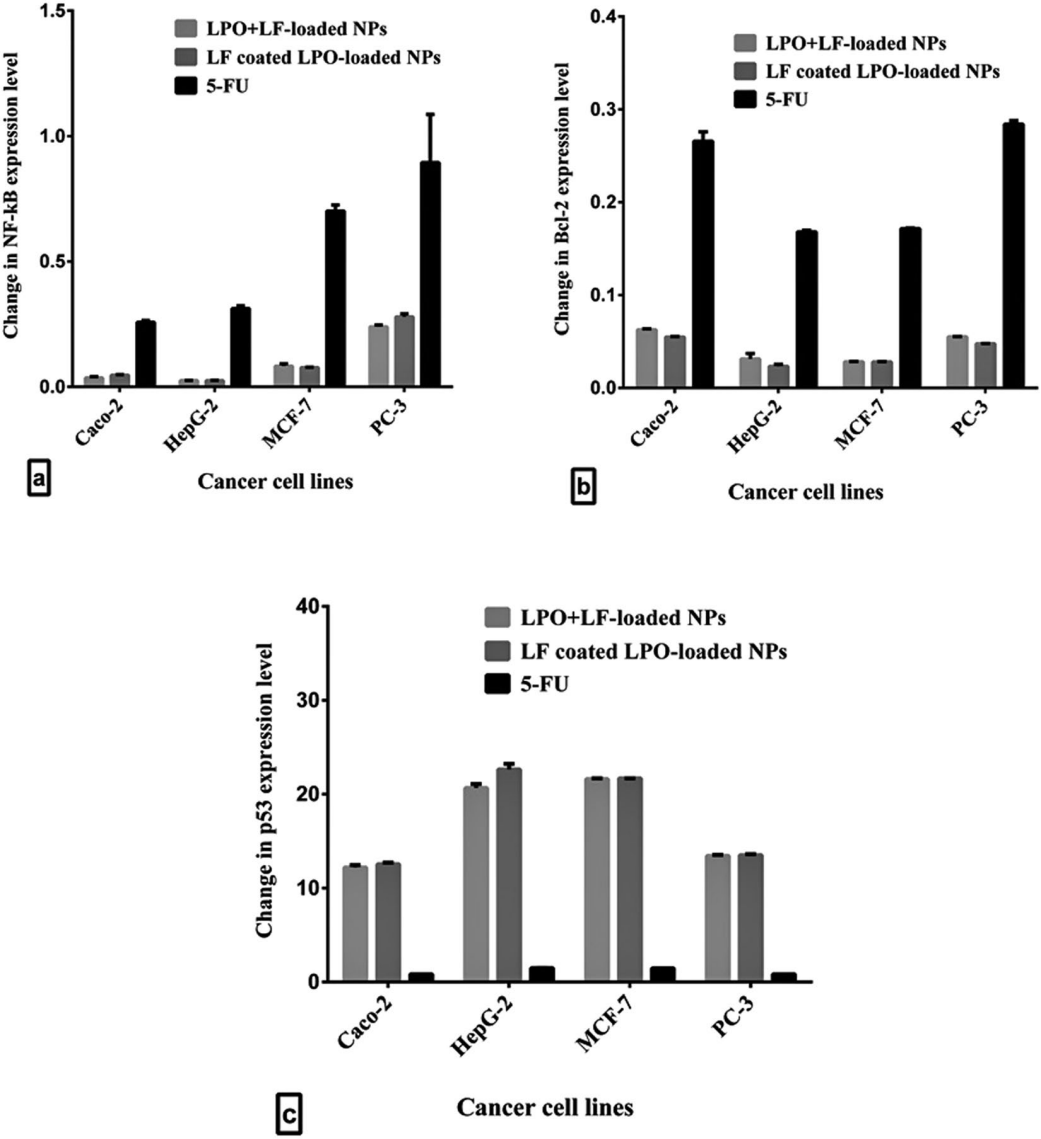
Relative changes in the expression levels of three key genes in human cancer cell lines after treatment with LPO + LF-loaded NPs, LF-coated LPO-loaded NPs and 5-FU for 72 h. (**a**) NF-κB. (**b**) Bcl-2. (**c**) p53.

Figure 6a demonstrates that NF-κB expression was suppressed in LF coated LPO-loaded NPs and LPO + LF-loaded NPs-treated Caco-2, HepG-2 and MCF-7 cells by more than 10 folds compared to 4, 3 and 1 folds, respectively, in 5-FU-treated cancer cells. While NF-κB mRNA level in PC-3 cell line was downregulated by about 4 folds compared to one fold in 5-FU-treated PC-3 cells. Subsequently, Bcl-2 expression level was significantly decreased in both NPs-treated cancer cells by more than 15 folds and 5 folds compared to untreated healthy cancer cells and 5-FU-treated cancer cells, respectively (Fig. 6b). In the same time, proapoptotic protein (p53) was markedly upregulated in NPs-treated cancer cells by more than 10 folds compared to untreated and 5-FU-treated cancer cell lines (Fig. 6c).

## Discussion

The purified milk proteins are considered promising anticancer drugs thus it is necessary to protect these proteins from the physiological barriers of the body and improve their delivery to tumor sites through nanoformulation of these proteins^21^ using chitosan. Chitosan is derived from a naturally occurring polymer and has tremendous compatibility with other ingredients owing to the existence of the great density of hydroxyl groups and amino groups in its polymer structure^22^. Due to its unique biological activities including broad antimicrobial^23,24^, immune-enhancing and anticancer effects^25,26^, chitosan has been utilized as a drug carrier with rare cytotoxic effects^27^. Chitosan NPs are characterized by their colloidal stability in biological fluids when carrying proteins^28^. Also, chitosan NPs were easily able to encapsulate large quantities of proteins (e.g., bovine serum albumin)^29^ and multiple anticancer drugs^30,31^. These chitosan properties are useful to exhibit high therapeutic effects and overcome drug resistance of cancer cells. Hence, we loaded and/or coated purified LF and LPO into chitosan NPs to investigate enhancing effect of LF on LPO anticancer activity and the intrinsic advantage of chitosan NPs on the stabilities and anticancer efficiency of LF and LPO.

Our data clearly revealed that the particle size and zeta potential of formulated chitosan NPs with LF and LPO were significantly affected by the coating or loading proteins processes. Generally, the sizes of loaded proteins to chitosan NPs were smaller than the sizes of coated proteins to chitosan NPs. As anticipated, the charges of all proteins-loaded chitosan NPs were more positive than proteins-coated NPs. The coating process was promoted as a function of charge density. The positive surface charge density of LPO and/or LF coated NPs was relatively lower than LPO and/or LF-loaded NPs due to the neutralization action by protein during the coating process. Furthermore, this charge neutralization of proteins decreased repulsion energy between NPs that increasingly assist agglomeration process and stimulate the creation of larger NPs^32^. These prepared chitosan NPs were also characterized by their appropriate loading (>50%) and desorption capacity (>80%) for the studied proteins.

The stability and activity of enzymes are mostly affected during immobilization dependent on the immobilization methods (e.g., entrapment and adsorption). Based on this fact, in this study, unlike LPO entrapment, LPO adsorption did not improve its stability and activity (CI > 1) may be due to alterations in its surface microenvironment and/or its physical and chemical properties. All of these may affect its kinetic parameters and maintaining the tertiary structure of substrate binding or active sites. Meanwhile, the loading of LPO may be done by interaction of reactive groups on chitosan and enzymatic residues that are away from the substrate binding and active sites and without changing its proper surface microenvironment^33^. Consequently, LPO-, LPO + LF-loaded NPs and LF coated LPO-loaded NPs as well as LF-coated NPs were able to retain LPO and LF activities for a longer time during storage compared to free and other nanoformulated forms. The stability and activity of enzymes and proteins formulated to chitosan-based NPs in different investigations have been established to be greater than their free form^28,34,35^. Our data were agreement with Nayeri *et al*.^36^ who found that LPO-encapsulated tragacanth-chitosan NPs is more stable than the free LPO under storage at +4 °C and 25 °C for 14 days and when heating from 0 to 80 °C.

These four more stable nanoformulated forms of LPO or LF showed high cytotoxic effect to human cancer cells (based on their low IC_50_ values and disrupted morphology of NPs-treated cancer cells) without adverse cytotoxic effect on the viability of human normal fibroblast cells. To the best of our knowledge, this study was the first to investigate enhancement of the anticancer activity of LPO and LF by loading or coating to chitosan NPs against different cancerous cell lines. We can deduce that LF coated LPO-loaded NPs and LPO + LF-loaded NPs were the most potent NPs against all tested cancerous cells with the highest selective and synergistic indexes. Hence coating or loading LF and encapsulating LPO into chitosan strongly boosted their anticancer activity to be significantly more effective with high synergism compared to free single or combined LPO and LF or other NPs. This is may be attributed to, firstly, the coated LPO forms failed to maintain LPO stability and activity in comparison with the loaded forms whereas no difference in LF activity between its loaded and coated forms. Secondly, the combination of LPO-loaded NPs with LF, by loading or coating, confers additional stability to LPO. This may be related to the antioxidant activity of LF that acts as an active protector to LPO against oxidative destruction by the generated free radicals in cancer cells^37,38^. This was confirmed by higher stability and activity of LPO + LF-loaded NPs and LF-coated LPO-loaded NPs compared to LPO-loaded NPs (Fig. 2a,b) and subsequently higher synergistic anticancer effect (Table 2 and Supplementary Table S2). Moreover, the cationic properties of these NPs give them remarkable ability to disrupt the cancer cell membrane and thereby are selective toxic against cancer cells without adversely affecting normal cells. Indeed, the cell membrane in cancer cells is more negatively charged than normal cells makes them more attractive to the cationic proteins and peptides, thus leading to cancer cell death and not normal cells death^39^.

In the current study, the synergistic apoptotic-mediated anticancer effect of both NPs (LF coated LPO-loaded NPs and LPO and LF-loaded NPs) was declared by high percentage of annexin-stained cells and sub G1 populations as well as orange fluorescence nuclear staining of treated cancer cells in comparison with untreated cancer cells. A previous study illustrated that lactoferricin B peptide fragment of LF induced caspase-dependent apoptosis of human gastric cancer cell line^40^. Another important finding in another study showed that LPO participated in apoptosis-dependent antineoplastic effect of iodine or iodide in mammary cancer^41^. In this study, the apoptotic mechanisms that may be behind the anticancer potential of both NPs were observed via suppression of NF-κB expression and consequently downregulation of Bcl-2 and upregulation of p53 in all treated cancer cell lines. Upon activation and nuclear translocation of NF-κB that is common in solid tumors^42^ as one of major stimulators of uncontrolled proliferation, prosurvival proteins (e.g. Bcl-2) are transcriptionally induced and p53 is inactivated via downregulation of p38 MAPK^43,44^. Compatible with our findings, recent studies have reported that bovine LF revealed anticancer potential against human colon and breast cancer cells by diverse signaling pathways, including Bcl-2, p53 and caspase-dependent apoptosis^45,46^. Moreover, Morita *et al*.^47^ found that LPO was able to downregulate NF-κB in an *in vitro* model of bone resorption. Furthermore, previous studies have reported the apoptosis-mediated anticancer effect of chitosan was associated with arresting G1/S cell cycle, increasing p53 level and decreasing Bcl-2 level against different cancer cell lines^48–50^. In addition, chitosan oligosaccharides inhibit activation of NF-κB as one of the mechanisms to reduce lipopolysaccharides-induced inflammation in Caco-2 cell line^51^.

Beside high porosity of chitosan NPs increases retention of LPO and LF, these NPs allow drugs (milk proteins) to directly and selectively interact with biomolecules of cancer cells by improving their intracellular delivery, subsequently causing selective severe alterations in cancer cells then death^52,53^. In addition to all these above mechanisms, both the immobilization using chitosan and loading or coating with LF enhance LPO features with respect to its highest stability and activity (Fig. 2a,b) that may interpret the synergy as net anticancer effect of combination between LPO, LF and chitosan.

Hence, this novel stable combination and nanoformulation of two milk proteins by coating or loading LF to LPO-loaded chitosan NPs revealed a significant synergistic induction of apoptosis in the different studied cancer cell lines. This occurs at low doses and high selectivity in comparison with free proteins (particularly, LPO), other tested NPs and currently used chemotherapy. These findings provide a support for future applications of coating or loading of LPO-loaded NPs with LF as potential therapeutic agents for different tumor types with minimum risk on normal cells.

## Materials and Methods

Bovine skim milk was prepared from raw milk according to the method of Almahdy *et al*.^54^. Bovine LPO and LF purifications were performed from the skimmed milk according to methods of El Fakharany *et al*.^55,56^ with some modifications. In brief, bovine milk was defatted by centrifugation at 5000 rpm for 30 min and decaseinated by decreasing the pH to 4.2 with 1 M HCl solution. The skimmed milk was obtained by centrifugation at 4000 rpm for 20 min and the supernatant was dialyzed overnight against 50 mM Tris HCl buffer, pH 7.6 (working buffer, WB). This skimmed milk was applied to the pre-equilibrated Mono S 5/50 GL column. LPO and LF were eluted with WB containing NaCl gradient of 0.0–1.0 M. After dialysis, the pooled fractions of LPO or LF were applied to a Sephacryl S100 column (5 × 150 mm, GE Health care, Sweden) equilibrated with WB and eluted with WB containing 150 mM NaCl. The purity and molecular weight of both LPO and LF were estimated by SDS-PAGE. Both purified LPO and LF fractions were pooled, dialyzed, lyophilized, and kept in −80 °C until use.

### Preparation of LPO and/or LF-loaded chitosan NPs and coated chitosan NPs with LPO and/or LF

Chitosan NPs were prepared according to ionic gelation method as described by Anitha *et al*.^57^ with our modification. Chitosan (2 mg/ml) was dissolved in 0.1% acetic acid with continuous stirring and pH was adjusted to be 5.5. Bovine LPO and/ or LF (0.5 mg/ml) were added drop wise over 60 min then dextran sodium sulfate (cross linker) was added slowly to obtain NPs. The formed NPs were collected by centrifugation twice at 12000 rpm for 40 min at 4 °C and the pellets were suspended in phosphate buffer saline (PBS). After lyophilization of these NPs, 10 mg of each prepared NPs were resuspended in 1 mM Tris-HCl buffer (pH 9) for the next coating step.

The coating was performed as described by Abu-Serie and El-Rashidy^52^. During stirring of the above prepared unloaded- or loaded-chitosan NPs, 4 mg/ml of LPO and/or LF were added drop wise. The coated NPs were then collected by centrifugation twice for 40 min at 12000 rpm at 4 °C, suspended in PBS and freeze-dried before further use.

### Characterization of the prepared nanoparticles

- Determination of LPO and/or LF encapsulation efficiency, loading and desorption capacity of loaded and coated NPs: The protein content in the supernatant during the NPs preparation was quantified by Bradford spectrophotometric method at 595 nm^58^. LPO and LF loading capacity (LC) and encapsulation efficiency (Encaps) of NPs were calculated as follow: %LC = [(A-B)/C] × 100 and %Encaps = [(A-B)/A] × 100, respectively. Where A is the total amount of protein, B is the free amount of protein in the supernatant and C is the weight of NPs. In addition, the percentage of protein desorption (% Des) during the coating procedure was estimated as % Encaps in the supernatant aliquot of washing step of coating.

- Detection of particle size and zeta potential of loaded and coated NPs was done using a Zetasizer Nano ZS (Malvern, UK).
- Morphological analysis of the most active NPs was determined by scanning electron microscopy (JEOL, model JSM-6460LV, Japan) at 20 kV.

### Determination of LPO and LF activities before and after nanoformulation (stability study)

The activity of free, loaded and coated LPO was determined throughout 9 weeks using 2,2′-Azino-bis(3-ethylbenzthiazoline-6-sulfonic acid (ABTS) as it was described by Shindler and Bardsley^59^. The LF activity in all free and nanoformulated samples was determined for 9 weeks according to the procedure of Ye *et al*. using nitroblue tetrazolium (NBT) reagent^60^. LPO and LF activity was expressed in IU per mg protein which was quantified by Bradford method^58^.

### Determination of cytotoxicity of protein samples against human normal and cancer cell lines

Free and nanoformulation of LPO, LF and mixture of LPO plus LF (1:1) as well as unloaded and uncoated chitosan NPs were tested for their cytotoxic effects on human cells using MTT according to Mosmann method^61^. Human normal dermal fibroblast cell line was cultured in supplemented DMEM medium (Lonza, USA) with 5% fetal bovine serum (FBS). Fibroblast cells (3 × 10^3^/well) were seeded in 96-well cell culture plate and allowed to attach for 24 h at 37 °C in 5% CO_2_ incubator. Then serial concentrations of LPO, LF, mixture of LPO plus LF, the prepared (loaded and coated) NPs and 5-flourouracil (5-FU) were added. After 72 h incubation in 5% CO_2_ incubator, MTT reagent (5 mg/ml) was added to each well and the plates were incubated at 37 °C for 3 h. Then MTT solution was removed, DMSO (150 µl) was added and the absorbance was measured with a microplate reader at 570 nm. The half maximal inhibitory concentration (IC_50_) and safe dose (EC_100_) values of all protein samples and 5-FU that cause 50% and 100% cell viability, respectively, were estimated by the Graphpad Instat software.

Anticancer effect of LPO, LF, mixture of LPO plus LF (1:1) and eight NPs was assayed using four human cancer cell lines in control with 5-FU (standard chemotherapy drug). Colon cancer cell line (Caco-2) and prostate cancer cell line (PC-3) were maintained as adherent cell cultures in DMEM containing 10% FBS (GIBCO, USA) while hepatoma cell line (HepG-2) and breast cancer cell line (MCF-7) were cultured in RPMI-1640 supplemented with 10% FBS. All cancer cell suspensions (3 × 10^3^ cells/well) were seeded into 96-well plates and allowed to attach for 24 h. Then serial concentrations of protein samples and 5-FU were incubated with four cancer cell lines at 37 °C in 5% CO_2_ incubator for 72 h. The sensitivity of tumor cells to the tested samples was evaluated using MTT assay (as described above). The IC_50_ values was calculated using Graphpad Instat software and the selectivity index (SI) that defined as the ratio of the IC_50_ on normal cells versus cancer cells was also estimated. The most effective anticancer samples were investigated, at their IC_50_ doses, by phase contrast microscope in comparison with untreated cells and 5-FU-treated cells.

The synergy between LPO and LF as well as between these protein and chitosan in the single and combined nanocombinations in the term of IC_50_ values was investigated by the estimation of combination index (CI) according to Long *et al*.^62^. CI that is <1 refers a synergistic effect while >1 indicates an antagonistic effect and =1 for an additive response.

### Analysis of cell death effect using flow cytometry and fluorescence phase contrast microscope

Human cancer cell lines (HepG-2, Caco-2, MCF-7 and PC-3) and normal cells (fibroblast) were treated with IC_50_ concentration of the most effective anticancer protein samples in control with IC_50_ of 5-FU. After incubation for 72 h, cells were trypsinized, washed with PBS and incubated with annexin V- biotin (Sigma, USA) and propidium iodide (PI) for 15 min. After staining, cells were fixed, washed with PBS and incubated with 5 µg/ml of streptavidine-fluorescein (Sigma, USA) for 15 min. The cell death rates were quantified by flow cytometry analysis (Ex = 488 nm; Em = 530 nm) using FITC signal detector (FL1) and phycoerythrin emission signal detector (FL2) for annexin and PI staining, respectively. The synergy between LPO and LF nanocombinations in the term of percentage of total apoptosis was detected by the calculation of CI that was obtained via dividing the excepted value of total apoptosis by the observed value of total apoptosis.

Additionally, antitumor activity of the most effective NPs, at IC_50_, was investigated under fluorescence phase contrast microscope after staining the treated cancer cells with double fluorescent nuclear dyes; ethidium bromide and acridine orange (Sigma, USA).

### Cell cycle distribution by flow cytometry

Change in cancer cell cycle distribution before and after treatment with IC_50_ of the most effective anticancer samples was determined by flow cytometry as described by Jass *et al*. method^63^. In brief, the untreated and treated cancer cells were incubated with 5 μg/ml RNase A (Sigma, USA) then mixed with 10 μl of 1 mg/ml PI (Sigma, USA) for flow cytometry analysis (Partec, Germany) at 488 nm using Cell Quist and Mod Fit softwares.

### Quantitative detection of the effect of prepared NPs on the expression of nuclear factor-kappa B (NF-κB), tumor suppressor gene (p53) and oncogene (Bcl-2) in cancer cells

Total RNAs of untreated and the two most effective anticancer NPs-treated cancer cells were extracted using Gene JET RNA Purification Kit (Thermo Scientific, USA). The cDNA was synthesized from mRNA using cDNA Synthesis Kit (Thermo Scientific, USA). Real time PCR was performed using SYBR green master mix and specific primers (Forward/Reverse) were 5′-TACTCTGGCGCAGAAATTAGGTC-3′/5′-CTGTCTCGGAGCTCGTCTATTTG-3′ for NF-kappa B gene. While 5′-TAACAGTTCCTGCATGGGCGGC-3′; 5′-AGGACAGGCACAAACACGCACC-3′ was for p53 gene and 5′-TCCGATCAGGAAGGCTAGAGTT-3′/5′-TCGGTCTCCTAAAAGCAGGC**-**3′ was for Bcl-2 gene. The 2^−ΔΔCT^ equation was used to estimate change in gene expressions of NF-kB, p53 and Bcl-2 before and after treatment of cancer cells.

### Statistical Analysis

Data (n = 5) were expressed as mean ± standard error of the mean (SEM). Statistical significance was estimated by the multiple comparisons Tukey post-hoc analysis of variance (ANOVA) using SPSS16 program. The differences were considered statistically significant at p < 0.05.

### Availability of data and materials

All data generated or analysed during this study are included in this published article

## Electronic supplementary material

Supplementary Tables
