# Supplementary material for: Efficiency of novel nanocombinations of bovine milk proteins (lactoperoxidase and lactoferrin) for combating different human cancer cell lines
Source: Sci Rep. 2017 Dec 1;7:16769. doi: 10.1038/s41598-017-16962-6 (PMC5711920; doi:10.1038/s41598-017-16962-6)
Supplement: Supplementary file 1 — Supplementary Tables [file 41598_2017_16962_MOESM1_ESM.pdf]

Efficiency of novel nanocombinations of bovine milk proteins (lactoperoxidase and lactoferrin) for combating different human cancer cell lines

Marwa M. Abu-Serie[1] & Esmail M. El-Fakharany[2]

[1]Medical Biotechnology Department, Genetic Engineering and Biotechnology Research Institute, City for Scientific Research and Technology Applications (SRTA-City), New Borg EL-Arab 21934, Alexandria, Egypt.

[2]Protein Research Department, Genetic Engineering and Biotechnology Research Institute, City for Scientific Research and Technology Applications (SRTA-City), New Borg EL-Arab 21934, Alexandria, Egypt.

Correspondence and requests for materials should be addressed to M.M.A. (email: marwaelhedaia@gmail.com) and E.M.E. (email: esmailelfakharany@yahoo.co.uk)

Table S1 IC_{50} (μg/ml) and EC_{100} (μg/ml) of all tested NPs samples against human normal cell line

Sample	Fibroblast	
	IC_{50}	EC_{100}
Free chitosan NPs (unloaded and uncoated NPs)	437.41±8.33	190.99±2.8
LPO	829.8±27	332.4±15.1
LPO-loaded NPs	2557.2±12	1388±9.3
LPO coated NPs	1208.3±83	414.4±12.5
LF	816.1±9.3	389.1±7.2
LF-loaded NPs	1357±50.3	469.6±6.2
LF coated NPs	2311.6±83.8	1244.7±14.4
LPO+LF	900.8±11.4	315.9±3.4
LPO+LF-loaded NPs	3283.2±35.1	1374.7±7.8
LPO+LF coated NPs	1102.3±57.8	396.7±89.1
LF coated LPO-loaded NPs	2116.3±14	1145.1±4.5
LPO coated LF-loaded NPs	999.7±4	334.4±2.5
5-FU	1.9±0.14	0.79±0.17

All values were expressed as mean±SEM.

Table S2 Combination cytotoxic effect of LPO, LF and chitosan in the prepared NPs samples against human cancer cell lines in the term of CI values

NPs samples	CI values			
	Caco-2	HepG-2	MCF-7	PC-3
LPO-loaded NPs	0.946±0.003	0.804±0.002	0.942±0.0006	0.858±0.001
LF-loaded NPs	0.843±0.001	0.861±0.0003	0.872±0.001	0.882±0.002
LF coated NPs	0.888±0.001	0.795±0.003	0.893±0.002	0.897±0.002
LPO+LF-loaded NPs	0.842±0.0001	0.748±0.001	0.836±0.0007	0.852±0.001
LPO+LF coated NPs	1.354±0.014	1.128±0.026	1.27±0.018	1.297±0.001
LPO coated LF-loaded NPs	1.254±0.007	1.155±0.01	1.061±0.001	1.216±0.002
LF coated LPO-loaded NPs	0.826±0.0008	0.706±0.0007	0.845±0.0001	0.837±0.005
LPO coated NPs	1.664±0.035	1.432±0.007	1.331±0.006	1.554±0.029

All values were expressed as mean±SEM.
